# Dietary Advanced Glycation End Products (AGEs) and Urinary Fluorescent AGEs in Children and Adolescents: Findings from the Italian I.Family Project

**DOI:** 10.3390/nu16121831

**Published:** 2024-06-11

**Authors:** Marika Dello Russo, Ivana Sirangelo, Fabio Lauria, Annarita Formisano, Clara Iannuzzi, Antje Hebestreit, Valeria Pala, Alfonso Siani, Paola Russo

**Affiliations:** 1 Institute of Food Sciences, National Research Council, 83100 Avellino, Italy; marika.dellorusso@isa.cnr.it (M.D.R.); annarita.formisano@isa.cnr.it (A.F.); alfonso.siani@isa.cnr.it (A.S.); paola.russo@isa.cnr.it (P.R.); 2 Department of Precision Medicine, University of Campania “L. Vanvitelli”, 80138 Naples, Italy; ivana.sirangelo@unicampania.it (I.S.); clara.iannuzzi@unicampania.it (C.I.); 3 Leibniz Institute for Prevention Research and Epidemiology—BIPS, 28359 Bremen, Germany; hebestr@leibniz-bips.de; 4 Epidemiology and Prevention Unit, Fondazione IRCCS, Istituto Nazionale dei Tumori, 20133 Milan, Italy; valeria.pala@istitutotumori.mi.it

**Keywords:** advanced glycation end products (AGEs), urinary AGEs, diet, children, I.Family project, ultra-processed foods (UPF), population study

## Abstract

Advanced glycation end products (AGEs) have been implicated in chronic diseases in adults, but their role in paediatric populations remains uncertain. This study, conducted on the Italian sample of the I.Family project, aimed to investigate the relationship between dietary and urinary fluorescent AGEs in children and adolescents. The secondary objective was to investigate the sources of dietary AGEs (dAGEs) and their association with dietary composition and anthropometric parameters. Dietary data were collected from 1048 participants via 24 h dietary recall in 2013/2014 to estimate dAGEs intake, while urinary fluorescent AGE levels were measured in 544 individuals. Participants were stratified based on dAGEs intake and compared with respect to urinary fluorescent AGE levels, anthropometric measurements, and dietary intake. The results showed no significant correlation between dietary and urinary fluorescent AGE levels, nor between dAGEs and anthropometric parameters. Notably, higher dAGEs were associated with a diet richer in protein (especially from meat sources) and fat and lower in carbohydrates. In addition, the consumption of ultra-processed foods was lower in participants with a higher DAGE intake. This study highlights the lack of a clear association between dietary and urinary fluorescent AGEs in children, but suggests a distinctive dietary pattern associated with increased dAGEs intake. Further investigation is warranted to elucidate the potential health implications of dAGEs in paediatric populations.

## 1. Introduction

Advanced glycation end products (AGEs) are a collection of heterogeneous compounds formed spontaneously when sugars undergo non-enzymatic and non-selective reactions with proteins or lipids, a phenomenon commonly known as the Maillard reaction or glycation [1,2,3].

The human body encounters AGEs from two main sources: endogenous AGEs, also known as biological AGEs, which are formed as by-products of glucose metabolism or lipid peroxidation, and exogenous AGEs, which are mainly found in food (dietary AGEs, dAGEs) and cigarette smoke [4]. While the formation of AGEs in the body is a physiological process, excessive production and accumulation in body tissues over a lifetime can contribute to adverse health outcomes [5,6]. The accumulation of AGEs in body tissues irreversibly accelerates cellular ageing and alters protein structure and function [5].

AGEs can be classified into fluorescent and nonfluorescent based on their ability to emit fluorescence and their chemical structure [3,6]. Among others, carboxymethyl-lysine (CML), carboxyethyl-lysine (CEL), pyrraline, pentosidine, methylglyoxal-lysine dimer (MOLD), and fluorescent AGEs are considered the key markers for AGEs due to their widespread occurrence and ease of detection in human fluids, tissues, and food [3].

Dietary AGEs (dAGEs) naturally occur in foods, but their rate of formation is markedly increased during cooking processes, particularly those involving high temperatures and dry heat, such as grilling, broiling, frying, or roasting [7]. This explains why processed foods like cookies, snacks, and processed meats, which are often consumed by children and adolescents, have high dAGE levels [4,7].

Excessive intake and accumulation of dAGEs have been implicated in the dramatic rise in the prevalence of non-communicable diseases, such as obesity, diabetes, atherosclerosis, as well as oxidative stress and inflammation [8,9,10]. A meta-analysis of prospective studies has also shown an increased risk of all-cause and cardiovascular mortality with higher circulating AGEs [11].

Moreover, animal studies have shown a correlation between a reduction in dAGEs and a significant decrease in circulating AGE levels, along with diseases associated with inflammation and oxidative stress [12]. While there is sufficient data to suggest a role for dAGEs in inducing low-grade chronic inflammation, oxidative stress, insulin resistance, and vascular dysfunction in adults, limited research has examined the effects of AGEs in children and adolescents, and mixed results have been obtained [13].

As it is evident that the accumulation of AGEs in the body may have harmful potential, it is also useful to investigate whether dAGEs contribute to the body’s AGEs pool. AGEs are absorbed in the digestive tract and circulate in the blood stream until they are subsequently excreted by the kidneys [14]. Monitoring urinary AGE excretion can be a valuable indicator of the body’s ability to eliminate these compounds, potentially providing insights into overall health and assessing disease risk [15]. In a previous study, we showed that measuring fluorescent AGEs in urine can serve as a simple, non-invasive, and early biomarker of subclinical inflammation in healthy children and adolescents [16].

Controversial data, however, exist regarding the association between dAGE consumption and plasma/serum and urinary AGEs. Some human and animal studies suggest a positive and independent correlation between dAGEs and AGE levels in body fluids [17,18,19]. Other authors have found no significant effect or even an inverse association of dAGEs intake and AGEs levels in serum, plasma, or urine [20,21,22].

Chronic exposure to high levels of dAGEs has been demonstrated to promote chronic inflammation and insulin resistance, both of which are underlying mechanisms of obesity and metabolic syndrome [23], although conclusive evidence of causality or consequentiality remains elusive. In addition, there is a paucity of studies investigating AGEs in children and adolescents, and the results of the available studies have been inconclusive [13].

In this study, our primary objective was to investigate the relationship between dAGEs and urinary fluorescent AGEs in Italian children and adolescents from the I.Family project. As secondary objectives, we sought to assess the sources of dAGEs and the association between dAGEs, nutritional composition of the diet, and anthropometric parameters in this population.

## 2. Materials and Methods

### 2.1. Study Population

**Figure 1:**
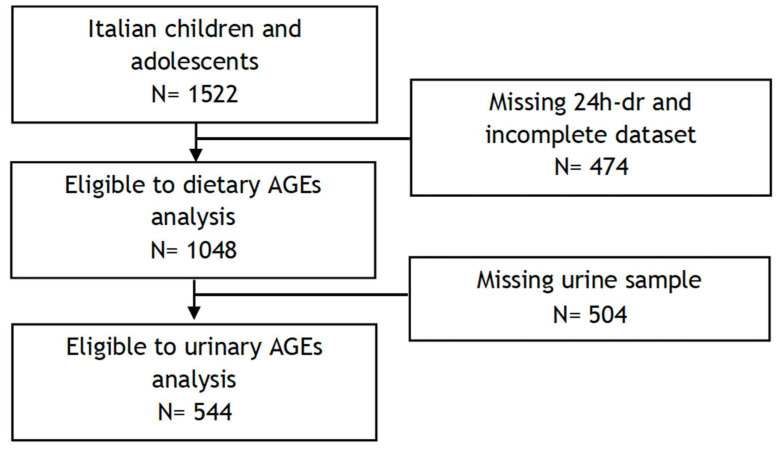
Flow chart of participants included in the final analyses.

The I.Family project (http://www.ifamilystudy.eu, accessed on 7 June 2024), aimed to assess the determinants of eating behaviour in children and adolescents from eight European countries and related health outcomes, was built on the IDEFICS study (http://www.ideficsstudy.eu, accessed on 7 June 2024), which was established in 2006 and followed up in 2013–2014 [24,25]. Briefly, the Italian cohort of the I.Family project was composed of 1522 children and adolescents who underwent a general examination module [25]. A total of 1048 children and adolescents, with available sociodemographic and anthropometric information and at least one completed 24 h dietary recall, were included in the analysis of dAGEs. Among them, 544 participants who provided a fasting urine sample for AGE determination were included in the analysis of urinary AGEs. The flow chart of the selection process is shown in Figure 1. Details of the general design, instruments, and survey characteristics can be found elsewhere [25].

Registration: The Pan-European IDEFICS/I.Family cohort is registered under ISRCTN62310987. Date assigned: 23 February 2018.

### 2.2. Ethics

The study was conducted in accordance with the Declaration of Helsinki and approved by the Ethics Committee of the local Health Authority (ASL Avellino), and informed written parental consent was obtained for each participant.

### 2.3. Physical Examination

A detailed description of the anthropometric measurements in the I.Family project, including details on intra- and inter-observer reliability, has previously been provided [26]. In brief, weight was measured using a body composition analyser (Tanita BC 420 SMA, Tanita Europe GmbH, Sindelfingen, Germany) to the nearest 0.1 kg. Height was determined using a calibrated stadiometer (Seca 225, Seca GmbH & Co., KG., Hamburg, Germany) with an approximation of 0.1 cm. Body mass index (BMI) was calculated by dividing body weight (in kg) by height squared (in m^2^), and z-scores specific to age and sex were calculated using the Cole and Lobstein method [27]. The classification of children and adolescents into normal weight, overweight, or obese categories was based on cut-offs provided by the International Obesity Task Force [27]. Waist circumference was assessed using an inelastic tape (Seca 200, Seca GmbH & Co., KG., Hamburg, Germany) with a range of 0–150 cm. Measurements were taken at the midpoint between the iliac crest and the lower border of the tenth rib while the subject was in a standing position, with arms relaxed at the sides. Measurements were recorded to the nearest 0.1 cm and z-scores specific to age and sex were calculated, according to Ahrens et al. [28]. Blood pressure (BP) was measured using an automatic device (Welch Allyn, Inc., 4200B-E2, Skaneateles Falls, NY, USA) with a cuff appropriate for the arm circumference. Measurements were taken after at least 5 min of rest in a seated position, following a standardized procedure [29]. Normalized (z-score) average systolic and diastolic blood pressure values, calculated according to Ahrens et al. [28], were used for statistical analysis.

### 2.4. Sample Processing and Analytical Procedures

Comprehensive details regarding sample collection and analytical procedures have previously been published [30]. During the first visit to the study centre, children, adolescents, or their parents were provided with a collection cup and instructions for obtaining morning urine samples. The morning urine was collected at home and promptly brought to the study centre on the same day (94% of the samples were first morning spot urine). No preservative was used, but parents were instructed to refrigerate the urine sample at home if the time between collection and delivery to the study centre exceeded two hours. Upon arrival at the study centre, urine samples were promptly frozen at −80 °C on the same day as collection.

### 2.5. Measurement of Urinary AGEs

Measurements of urinary fluorescent AGEs were performed on a Perkin Elmer Life-Sciences LS 55 spectrofluorimeter (PerkinElmer, Waltham, MA, USA), as previously described [16]. Urine samples were diluted at 1:10 in phosphate-buffered saline, and fluorescence spectra were recorded between 400 nm and 600 nm, upon excitation at 370 nm, at room temperature. The fluorescence intensity was measured in correspondence with the emission maximum centred at 440 nm and was corrected by subtracting the background. Evidence from a previous study showed that, for this fluorescence assay, the intra-assay coefficient of variation (CV) was 5.5% to 8.2%, and the inter-assay CV was 8.l% to 9.7% [31]. As the urinary AGE concentration depends on the urine volume, the relative fluorescence intensity (expressed in arbitrary units, AU) was adjusted for the urinary creatinine concentration, expressed as g/L. Urinary creatinine was measured by a colorimetric assay based on Jaffe’s reaction (COBAS INTEGRA 400 plus, Roche Diagnostics Ltd., CH-6343 Rotkreuz, Switzerland).

### 2.6. Socio-Demographic Data

Socio-demographic data were obtained through a validated and reproducible questionnaire. Adolescents completed their own questionnaires, while parents of younger children (i.e., aged < 12 years) provided proxy reports. Parents self-reported their highest educational level, classified according to the International Standard Classification of Education (ISCED) into three main categories: low (ISCED levels 1 and 2), medium (ISCED levels 3 and 4), and high (ISCED level 5) [32].

### 2.7. Dietary Data

Dietary data were gathered through web-assisted 24 h dietary recall (24-HDR), known as SACANA (Self-Administered Children, Adolescents, and Adult Nutrition Assessment). This 24-HDR has undergone validation as a self-reporting instrument for assessing dietary intake in children, adolescents, and adults [33,34]. A full description of the SACANA software can be found elsewhere [35]. The first 24-HDR was carried out at the examination centre. Subsequently, participants were instructed to complete two additional 24-HDRs on non-consecutive days, including one weekend day, over the next two weeks. Participants with at least one completed 24-HDR were included in our study. Parents were requested to assist younger children (<12 years) in completing their 24-HDRs. Participants reported details on the quantity and type of foods and drinks consumed during the preceding day, beginning from the first intake upon waking in the morning. Standardized photographs depicting foods in various portion sizes were utilized to aid in estimating the foods and beverages consumed [35]. To estimate the ultra-processed food (UPF) intake, each food and beverage reported in the 24-HDR interview was classified according to the NOVA classification [36] on the basis of the extent and purpose of industrial food processing. The relative contribution of UPFs to the total energy intake for each participant was computed and divided into age- and sex-specific quintiles. A detailed description of the UPF calculation can be found in the study of Lauria et al. [37].

### 2.8. Dietary AGEs

To assess the dietary intake of AGEs, data collected from the first 24-HDR interview were utilized. For each food and beverage reported in the 24-HDR, a value in kilounits per day (kU/day) for the estimated amount of AGEs was assigned using a published database of AGE content, which included 549 foods measured with a validated, non–cross-reactive monoclonal antibody [38]. When the AGE value was unavailable for a specific food or recipe, values from similar available foods or recipes were utilized. In cases where recipes were not found in the database, and AGE values for all ingredients were available, we assigned values to individual food components, considering the portion of each, and calculated the overall value of the recipe. The process of linking AGE values to the SACANA database was performed by one researcher and independently verified by another researcher. Any inaccuracies were addressed in a second review by a third researcher to prevent and minimise bias. The study also investigated dietary sources of dAGEs by categorizing foods based on their contribution to the typical Italian diet and culinary practices. Food groups were established to reflect commonly consumed items and cooking methods. These categories included the following. Proteic dishes non-UPFs: This category encompassed non-processed or minimally processed protein sources like meat or fish. Proteic dishes UPFs: This group included highly processed protein-containing items such as cheese-stuffed rolls, breaded chicken with spinach, toast, cheeseburgers, burger buns, sausages, and industrial cheese. Sweet snacks UPFs: This category comprised commercially produced sweet treats like cakes, candies, chocolate bars, spreads, and cookies. Salty snack UPFs: This group included chips, salty biscuits, and commercially produced bread. Single-item traditional dishes: popular Italian dishes as individual entities, such as pizza, lasagna (a meat, tomato sauce, cheese, and sometimes vegetable pasta dish) and parmigiana (breaded and fried eggplant layered with tomato sauce and cheese). This categorisation approach aimed to capture the unique dietary patterns and food processing methods relevant to the Italian population in the context of the dAGE intake assessment.

### 2.9. Statistical Analysis

Both dietary intakes and urinary levels of AGEs were not normally distributed. Consequently, the analyses are presented based on natural log (ln)-transformed data. Participants were categorized according to tertile cut-off points for their dietary intake. Total energy intake was adjusted using the residual method [39], whereby standardized residuals were computed by regressing the natural logarithm of AGEs on total energy intake, sex, and age for each participant. Dietary AGEs and nutrients were expressed as intake per 1000 kcal. In the descriptive analysis, continuous variables were presented as mean (M) and standard deviation (SD), while categorical variables were expressed as counts and percentages (%). Descriptive characteristics of the population were examined using one-way analysis of variance (ANOVA) for continuous variables and chi-square tests for categorical variables. One-way analysis of variance (General Linear Model) and multiple comparisons with Bonferroni’s correction were employed to assess the diet quality composition and health status across tertiles of dAGEs. Variables were expressed as mean and 95% confidence intervals (95% CI). When appropriate, analyses were adjusted for covariates including sex, age, family ISCED, and BMI. IBM SPSS Statistics (Version 23.0, IBM Corp., Armonk, NY, USA) was used for the statistical analyses, and statistical significance was set at a *p*-value less than 0.05.

## 3. Results

Table 1 displays the characteristics of the participants categorized by tertiles of dAGEs. Anthropometric parameters, prevalence of overweight/obesity, blood pressure levels, and sociodemographic characteristics were consistent across dAGEs tertiles. In the subgroup of participants who provided urinary samples, no significant differences in urinary fluorescent AGEs were observed across dAGEs tertiles.

As shown in Table 2, linear regression analysis, adjusted for covariates, did not reveal any association between dietary and urinary fluorescent AGEs. The linear regression analysis showed that only age significantly affected the variability of urinary fluorescent AGEs, with no significant association observed with dAGEs.

We evaluated the nutritional characteristics of the diet across dAGE tertiles (Table 3). Energy intake did not differ between dAGEstertiles. Regarding macronutrients, there was a significant positive trend for protein and fat intake across dAGE tertiles, while an inverse trend was observed for carbohydrate intake. Saturated fatty acid intake was significantly lower in the lower dAGE tertile than in the other two tertiles. No differences were found between the dAGE tertiles for sugar and fibre intakes. When considering energy intake from UPFs, a significantly lower intake was observed in the higher dAGE tertile compared to the other two tertiles.

Finally, the contributions of different foods and food groups to total dAGEs were analysed. As shown in Table 4, about half of the dAGEs intake came from non-UPF protein dishes (43%), especially from meat and breaded meat. In the third dAGEs tertile, the contributions of meat and breaded meat were 25.2% and 15.9%, respectively, significantly higher than in the other two tertiles. There was a significant negative trend in the contribution of fish, cheese, eggs, and processed meat to dAGEs intake. Other relevant contributors to daily dAGEs in our population were proteic UPF dishes (12.6%) and pizza (12.0%). The latter accounted for 20.8% of the dAGEs intake in the third dAGEs tertile. Lasagna accounted for 5.6% of dAGEs in the higher dAGEs tertile, significantly higher than the other two. The contribution of pasta/cereal dishes, snacks, and sauces, both UPF and non-UPF, to dAGEs intake showed a significantly negative trend.

## 4. Discussion

The relationship between dAGEs and their levels in plasma and urine is an important but poorly understood area of research, particularly with regard to its implications for health and disease management. The first aim of the present study was to investigate the relationship between dAGEs and urinary fluorescent AGE levels in a large sample of Italian children and adolescents. The analysis found no relationship between these two parameters, and dAGEs also showed no correlation with body measurements.

Previous studies on the relationship between dAGEs and urinary AGEs excretion have provided conflicting data.

There is evidence to suggest an association between dAGEs and urinary AGEs. Scheijen et al. showed that q higher intake of dietary AGEs is associated with significantly higher levels of AGEs in both plasma and urine. This association was found for the AGEs Nε-(carboxymethyl)lysine (CML), Nε-(1-carboxyethyl)lysine (CEL), and Nδ-(5-hydro-5-methyl-4-imidazolon-2-yl)-ornithine (MG-H1)1 [18].

An animal study investigating the relationship between dAGEs intake and urinary excretion in domestic cats found significant positive relationships between intake and urinary excretion of CML, CEL, lysinoalanine (LAL), and pyrraline (PYR). This suggests that dietary AGEs are absorbed and excreted via the urinary system [19].

On the other hand, other studies have suggested a minimal impact of dietary AGEs on urinary AGEs. High consumption of AGE-rich foods is not a major determinant of serum and urinary carboxymethyl-lysine levels in healthy adults [21]. In elderly T2DM patients with diabetic kidney disease, a low-AGE diet did not significantly affect the AGE content of skin or urine [22]. In non-diabetic individuals, no association was found between dAGEs and serum and urinary AGEs [20]. The conflicting data found in the literature are likely due to the significant variability in oral absorption and renal clearance kinetics of dAGEs under different health and disease conditions. According to Koshinsky et al., approximately one-third of AGEs are excreted in the urine in healthy individuals, whereas in individuals with diseases such as diabetes mellitus, less than 5% are excreted [40]. Of interest, the present study is the first to investigate the possible association of dAGEs and urinary AGEs in healthy children and adolescents.

Although dAGEs are a significant contributor to the body’s AGE pool, evidence on their metabolic fate and impact on health remains insufficient [23,41,42].

Endogenous AGE production within the body might be a more significant contributor to total AGE burden compared to dietary intake. It is plausible that non-dietary sources of AGEs could be a more pertinent risk factor, especially among the healthy and young population. Further research is needed to elucidate the relative contributions of these sources to health outcomes, particularly in younger individuals [43].

This study also examined the association between dAGE intake and nutrient composition. Key findings revealed no significant difference in total calorie intake between groups with varying dAGE levels. However, the macronutrient distribution differed. Individuals with higher dAGEs intake consumed a greater proportion of protein and fat, while their carbohydrate intake was lower. Interestingly, saturated fat intake was lower in those consuming fewer dAGEs. No significant variations were observed in sugar or fibre intake across dAGE intake levels.

The primary sources of dAGEs in this population consisted of non-ultra-processed protein sources (43% contribution), with meat, particularly breaded varieties, being the major contributor. The high-dAGE group exhibited significantly higher consumption of meat and breaded meat products. Other protein sources like fish, cheese, eggs, and processed meat contributed less to dAGE intake, with this contribution further decreasing in the lower dAGE intake group. Proteic ultra-processed foods and pizza were secondary contributors, with pizza intake being significantly higher in the high-dAGEs group. Finally, pasta/cereal dishes, snacks, and sauces (both ultra-processed and non-ultra-processed) displayed a decreasing trend of dAGEs contribution with lower overall intake.

In essence, children and adolescents with higher dAGEs intake exhibited dietary patterns characterized by increased protein and fat consumption, especially from meat sources, accompanied by lower carbohydrate and saturated fat intake. This pattern was also associated with a lower overall intake of ultra-processed foods. Notably, the intake of AGEs depends not only on the type and quantity of raw foods consumed by an individual, but also, and likely to a greater extent, on the thermal processing and cooking methods used [10].

Our dietary findings are consistent with the observations reported by Koyama et al. [44] in their large-scale investigation of American adults with diabetes, which utilized a similar methodological approach. Employing a distinct dAGE database [45], Cordova et al. [46] identified cereals or cereal products, meat and processed meat products, cakes and biscuits, dairy products, sugar and confectionery products, and fish and shellfish as the primary dietary sources of dAGEs in a sizeable European adult population.

Our findings do not demonstrate a statistically significant correlation between dAGEs and anthropometric measurements in this paediatric population. This observation aligns with the hypothesis that dAGEs may not be a primary aetiological factor in childhood obesity. It is noteworthy that previous investigations into the association between dAGEs and body mass and/or metabolic abnormalities have exclusively recruited adult participants [8,47,48,49,50].

Additionally, studies examining serum AGE levels in children have yielded results that diverge from those observed in adults. Sebekova et al. [51] reported lower serum AGE levels in obese children compared to their lean counterparts. Similarly, Accacha et al. [52] conducted a cross-sectional study and observed an inverse association between carboxymethyl-lysine (CML) levels, adiposity, and inflammatory markers in a cohort of obese middle-school children.

The influence of dAGEs on obesity and its associated complications remain understudied. While a direct causative role of AGEs in obesity initiation is unlikely, their potential contribution to disease progression and severity warrants further investigation [41].

Our study possesses inherent limitations alongside its strengths. Quantifying dAGEs presents intrinsic challenges. Given the critical role of meat and fat content in influencing average dAGE intake, accurate serving size assessment emerges as a crucial factor for determining dAGE exposure. While existing databases offer a comprehensive inventory of dAGE content in various food and beverage items [38,45], limitations exist regarding their inclusivity of all food items and the influence of diverse cooking methods. Furthermore, the dAGE database employed in this study pertains to a North American urban population [38], potentially leading to an incomplete representation of the dietary patterns within our study population.

The estimation of fluorescent AGEs in urine, while non-invasive and suitable for research purposes [3,16], possesses significant drawbacks. One limitation is its inability to identify specific molecules responsible for the fluorescent signal. Additionally, it quantifies only fluorescent AGEs, neglecting non-fluorescent AGEs, carboxymethyl-lysine (CML), and Nε-(carboxymethyl) lysine (MG-H1) present in urine, potentially underestimating the total urinary AGE burden [3]. Finally, the cross-sectional design of our study restricts the demonstration of any causal pathogenetic mechanisms.

The study benefits from a large sample size with comprehensive dietary, socioeconomic, and anthropometric data. Furthermore, the use of meticulously standardized phenotypic measurements strengthens the analysis. In fact, all measurements were conducted according to detailed standard operating procedures reported elsewhere [53]. Additionally, employing 24 h dietary recalls to assess AGE intake likely yielded a more accurate representation of dietary consumption. It is worth noting that the participants provided urine samples and dietary data on the same day.

## 5. Conclusions

In summary, this study investigated the relationship between dAGE intake and urinary fluorescent AGEs in a paediatric population. It found no significant correlation, suggesting a disconnect between dietary and urinary AGEs in children. This aligns with conflicting data from previous adult studies, highlighting the need for further research on dAGEs in younger populations. Interestingly, the study revealed a dietary pattern associated with higher dAGE intake. Children consuming more dAGEs had higher protein and fat intakes, including saturated fat, particularly from meat sources, and lower carbohydrate intakes. This pattern also involved less consumption of ultra-processed foods. The study also found no association between dAGE intake and body measurements, suggesting that dAGEs might not be a primary factor in childhood obesity. Overall, the study highlights the complexities of dAGEs in children. Future prospective studies and randomized controlled trials should investigate the potential association between dAGEs and adverse outcomes during growth, such as increased susceptibility to obesity and related metabolic disorders. This research could eventually inform new dietary recommendations for the general population and specific age groups.

## Figures and Tables

**Table 1:** Characteristics of participants according to dietary AGE tertiles (kU/1000 kcal).

	dAGEs Tertiles		
	I (*n* = 349)	II (*n* = 350)	III (*n* = 349)
dAGEs (kU/day)	6942 ± 3910	14,002 ± 5596	28,412 ± 41,547
dAGEs (kU/1000 kcal/day)	3675 ± 1342	7599 ± 1212	17,322 ± 38,713
Age (years)	11.6 ± 1.9	11.6 ± 2.1	11.6 ± 2.1
Sex (Female%)	50.1	50.3	47.3
ISCED			
Low	13.5	17.1	17.6
Medium	66.9	62.3	62.4
High	19.6	20.7	20.0
BMI z-score	1.20 ± 1.01	1.17 ± 1.05	1.19 ± 1.10
WC z-score	1.34 ± 1.19	1.36 ± 1.23	1.36 ± 1.24
Overweight/obese %	50.1	45.4	50.1
SBP z-score	0.21 ± 0.83	0.18 ± 0.76	0.16 ± 0.76
DBP z-score	0.24 ± 0.58	0.27 ± 0.56	0.22 ± 0.52
	(*n* = 187)	(*n* = 178)	(*n* = 179)
Urinary AGEs (AU)	303 ± 132	301 ± 108	298 ± 122

Values are expressed as mean ± SD. dAGEs (kU/day): natural log (ln)-transformed daily intake of AGEs; dAGEs (kU/1000 kcal/day): energy adjusted dietary intakes of AGEs; ISCED: International Standard Classification of Education; BMI: body mass index; WC: waist circumference; SBP: systolic blood pressure; DBP: diastolic blood pressure. BMI, WC, SBP, and DBP are expressed as age- and sex-specific z-scores. AU: arbitrary units.

**Table 2:** Linear regression analysis model with urinary fluorescent AGEs as a dependent variable.

Dependent Variable	Independent Variables	B (SE)	*p*-Value
Urinary AGEs (AU)	Age (years)	−24.11 (3.06)	<0.0001
	Sex (m/f)	11.56 (9.93)	ns
	BMI z-score	−7.94 (4.68)	ns
	dAGEs (category)	−1.08 (6.04)	ns

SE: standard error; AU: arbitrary units; BMI: body mass index; BMI z-score: Age- and sex-specific BMI z-scores; dAGEs (category): tertiles of dietary intakes of AGEs; ns: not significant.

**Table 3:** Energy, nutrients, and UPF intakes according to dAGE tertiles (kU/1000 kcal/day).

	dAGE Tertiles (kU/1000 kcal)		
	I (*n* = 349)	II (*n* = 350)	III (*n* = 349)
Energy (kcal/day)	1807 (1716–1898)	1790 (1705–1875)	1778 (1690–1866)
Protein (g/1000 kcal)	34.1 (32.7–35.6) ^a,c^	38.5 (37.1–39.8) ^b,c^	42.7 (41.3–44.1) ^a,b^
Fat (g/1000 kcal)	27.6 (26.2–29.0) ^a,c^	32.5 (31.1–33.8) ^b,c^	35.0 (33.6–36.3) ^a,b^
SFA (g/1000 kcal)	10.2 (9.6–10.9) ^a,c^	12.0 (11.4–12.6) ^c^	12.0 (11.4–12.6) ^a^
CHO (g/1000 kcal)	151.1 (147.7–154.6) ^a,c^	137.1 (133.9–140.3) ^b,c^	126.9 (123.6–130.2) ^a,b^
Sugars (g/1000 kcal)	38.5 (36.2–40.8)	41.1 (38.9–43.2)	41.5 (39.3–43.7)
Fibre (g/1000 kcal)	7.9 (7.5–8.3)	7.8 (7.4–8.1)	7.5 (7.1–7.9)
UPF (%TEI)	42.3 (40.0–44.7) ^a^	44.1 (41.8–46.3) ^b^	38.0 (35.7–40.3) ^a,b^

Analysis adjusted for sex, age, family ISCED, and BMI. Values are expressed as mean (95% CI). dAGEs (kU/1000 kcal/day): energy-adjusted dietary intakes of AGEs. SFA: saturated fatty acids; CHO: total carbohydrates; UPFs: ultra-processed foods; UPFs (%TEI): percentage of total daily energy intake from UPFs. For each nutrient, superscript lowercase letters in the same row indicate significant differences among tertiles: a = III vs. I; b = III vs. II; c = II vs. I.

**Table 4:** Percentage contribution of different foods to total dAGEs, according to dAGE tertiles.

		dAGEs Tertiles (kU/1000 kcal)			
	All (*n* = 1048)	I (*n* = 349)	II (*n* = 350)	III (*n* = 349)	*p*-Value
Proteic dishes (non-UPFs)	43.1 ± 32.3	37.1 ± 29.0	45.6 ± 31.9	46.6 ± 35.0	<0.001
Meat	21.6 ± 29.8	11.9 ± 22.8	27.8 ± 30.9	25.2 ± 32.3	<0.001
Breaded meat	7.2 ± 21.0	0.4 ± 5.4	5.4 ± 18.6	15.9 ± 28.7	<0.001
Fish	2.2 ± 9.2	4.7 ± 14.0	1.2 ± 5.9	0.7 ± 3.7	<0.001
Cheese	7.3 ± 15.3	11.7 ± 19.4	6.6 ± 13.7	3.6 ± 10.2	<0.001
Parmigiana	0.9 ± 7.2	0.8 ± 6.8	1.5 ± 9.9	0.4 ± 3.0	ns
Eggs	0.8 ± 5.1	1.2 ± 6.0	1.0 ± 6.3	0.2 ± 1.5	0.005
Processed meat	3.0 ± 7.9	6.2 ± 12.3	2.2 ± 4.2	0.7 ± 1.8	<0.001
Proteic dishes (UPFs)	12.6 ± 24.2	10.7 ± 22.8	14.0 ± 24.6	13.1 ± 25.2	ns
Pizza	12.0 ± 25.4	3.0 ± 13.1	12.1 ± 25.1	20.8 ± 31.2	<0.001
Pasta/cereal-based dishes	5.5 ± 10.1	9.6 ± 14.1	4.6 ± 7.7	2.2 ± 4.5	<0.001
Lasagna	2.6 ± 12.6	0.2 ± 3.8	1.8 ± 10.8	5.6 ± 18.2	<0.001
Filled pasta (UPFs)	0.9 ± 6.3	1.0 ± 8.0	1.1 ± 6.3	0.8 ± 3.8	ns
Potatoes	1.0 ± 5.8	1.2 ± 7.4	1.3 ± 6.3	0.5 ± 2.6	ns
Bread	0.4 ± 2.5	0.8 ± 4.2	0.2 ± 1.0	0.1 ± 0.3	<0.001
Crackers (UPFs)	1.0 ± 4.8	2.2 ± 8.0	0.6 ± 1.2	0.3 ± 0.7	<0.001
Salty pies	1.1 ± 6.3	2.2 ± 10.0	0.7 ± 3.6	0.4 ± 1.7	<0.001
Snacks (UPFs)	5.6 ± 11.7	7.6 ± 13.8	5.6 ± 10.8	3.8 ± 9.8	<0.001
Salty snack (UPFs)	1.5 ± 5.3	2.4 ± 6.9	1.2 ± 3.5	1.0 ± 4.7	<0.001
Sweet snack (UPFs)	4.1 ± 10.5	5.2 ± 12.3	4.3 ± 10.3	2.8 ± 8.6	<0.001
Cakes/sweets (not UPFs)	5.5 ± 10.3	10.1 ± 15.2	4.4 ± 6.3	2.1 ± 3.8	<0.001
Cookies (not UPFs)	3.2 ± 7.8	6.1 ± 12.0	2.5 ± 4.5	1.1 ± 2.2	<0.001
Bakery Brioches/croissant	1.0 ± 4.2	1.9 ± 6.7	0.7 ± 2.0	0.4 ± 1.2	<0.001
Handmade/bakery sweets	1.3 ± 5.5	2.1 ± 8.0	1.2 ± 4.2	0.6 ± 3.0	<0.001
Sauces (UPFs)	0.3 ± 2.0	0.3 ± 2.4	0.4 ± 1.9	0.3 ± 1.7	<0.001
Sauces not (UPFs)	3.3 ± 6.8	5.8 ± 9.7	3.0 ± 5.4	1.0 ± 2.2	<0.001
Oil	2.1 ± 5.3	3.1 ± 7.6	2.0 ± 4.2	1.0 ± 2.4	<0.001
Butter/cream	0.6 ± 4.6	1.1 ± 7.0	0.5 ± 3.0	0.3 ± 2.4	0.02
Nuts/dried fruits	0.6 ± 3.2	0.7 ± 3.7	0.7 ± 3.5	0.3 ± 2.0	ns
Vegetables	1.6 ± 6.1	2.7 ± 8.4	1.3 ± 5.2	0.8 ± 3.3	<0.001
Fruit	0.10 ± 0.35	0.20 ± 0.57	0.07 ± 0.14	0.03 ± 0.07	<0.001
Soft drinks	0.06 ± 0.18	0.12 ± 0.29	0.05 ± 0.08	0.03 ± 0.05	<0.001
Milk and yogurt	0.04 ± 0.08	0.07 ± 0.13	0.02 ± 0.03	0.01 ± 0.02	<0.001

Values are expressed as mean ± SD. dAGEs (kU/1000 kcal/day): energy-adjusted dietary intakes of AGEs; UPFs: ultra-processed Foods; proteic dishes non-UPFs: non-processed or minimally processed protein sources like meat or fish. Proteic dishes UPFs: highly processed protein-containing items such as cheese-stuffed rolls, breaded chicken with spinach, toast, cheeseburgers, burger buns, sausages, and industrial cheese. Salty snack UPFs: chips, salty biscuits, and commercially produced bread. Sweet snack UPFs: commercially produced sweet treats like cakes, candies, chocolate bars, spreads, and cookies. Single-item traditional dishes: pizza, lasagna (a pasta dish with meat, tomato sauce, cheese, and sometimes vegetables) and parmigiana (breaded and fried eggplant layered with tomato sauce and cheese); ns: not significant.

## Data Availability

All data produced or analysed during this study are included in this article.
